# Supplementary material for: Constructing HIV/AIDS in Chinese media (2010–2024): A mixed-methods study
Source: PLoS One. 2026 May 13;21(5):e0349242. doi: 10.1371/journal.pone.0349242 (PMC13170867; doi:10.1371/journal.pone.0349242)
Supplement: Attachment — Submitted filename: Response to Reviewers.docx [file pone.0349242.s002.docx]

**Dear Editor,**

Thank you for your prompt handling of our manuscript. We have made the revisions requested in your email in accordance with PLOS ONE’s requirements. We are grateful for the editor's valuable feedback.

1. Please ensure that your manuscript meets PLOS ONE's style requirements, including those for file naming.

**Response:**

We have formatted the manuscript according to the PLOS ONE style guide you provided.

2. Please note that PLOS One has specific guidelines on code sharing for submissions in which author-generated code underpins the findings in the manuscript. In these cases, we expect all author-generated code to be made available without restrictions upon publication of the work. Please review our guidelines at https://journals.plos.org/plosone/s/materials-and-software-sharing#loc-sharing-code and ensure that your code is shared in a way that follows best practice and facilitates reproducibility and reuse.

**Response:**

We have provided a public code link in the new Data Availability Statement.

New Data Availability Statement:

All relevant data are within the paper and its Supporting Information files. The code used in this study is available in the Figshare repository: <https://doi.org/10.6084/m9.figshare.30985558.>

**3.** Please upload a new copy of Figure 1 as the detail is not clear. Please follow the link for more information:  https://journals.plos.org/plosone/s/figures

**Response:**

We have uploaded a new, higher-resolution version of Figure 1 to ensure its clarity and detail are sufficient for the reviewers and readers.

**4.** Thank you for providing your underlying data as Supporting Information.

We note that the data set contains text or data that is not in English. Please note that PLOS is an English-language publisher, so we require data sets to be provided in English as well. Please upload an English-language version of your data set.

This will also allow us to determine if your data follows PLOS standards per our Data Availability policy here: <https://journals.plos.org/plosone/s/data-availability>

**5.** We note that there is identifying data in the Supporting Information file <AIDS Corpus.xlsx>. Due to the inclusion of these potentially identifying data, we have removed this file from your file inventory. Prior to sharing human research participant data, authors should consult with an ethics committee to ensure data are shared in accordance with participant consent and all applicable local laws.

Data sharing should never compromise participant privacy. It is therefore not appropriate to publicly share personally identifiable data on human research participants. The following are examples of data that should not be shared:

-Name, initials, physical address
-Ages more specific than whole numbers
-Internet protocol (IP) address
-Specific dates (birth dates, death dates, examination dates, etc.)
-Contact information such as phone number or email address
-Location data
-ID numbers that seem specific (long numbers, include initials, titled “Hospital ID”) rather than random (small numbers in numerical order)

Data that are not directly identifying may also be inappropriate to share, as in combination they can become identifying. For example, data collected from a small group of participants, vulnerable populations, or private groups should not be shared if they involve indirect identifiers (such as sex, ethnicity, location, etc.) that may risk the identification of study participants.

Additional guidance on preparing raw data for publication can be found in our Data Policy (https://journals.plos.org/plosone/s/data-availability#loc-human-research-participant-data-and-other-sensitive-data) and in the following article: http://www.bmj.com/content/340/bmj.c181.long.

Please remove or anonymize all personal information (Names), ensure that the data shared are in accordance with participant consent, and re-upload a fully anonymized data set. Please note that spreadsheet columns with personal information must be removed and not hidden as all hidden columns will appear in the published file.

**Response:**

Regarding Questions 4 and 5, we have addressed them together. After carefully reviewing your journal's Data Availability Guidelines and referring to the practices of similar studies published in your journal (e.g., Lu et al. 2023 ([https://doi.org/10.1371/journal.pone.0291862)),](https://doi.org/10.1371/journal.pone.0291862)),) we have modified the format of the Supporting Information. Specifically, we have changed it to present the original semantic matrices for all news texts (including the document-topic matrix and the topic-word list). This approach completely avoids the leakage of sensitive information and is sufficient to support the reproducibility of the research findings. Furthermore, we have translated all Supporting Information into English to facilitate understanding by the target readership.

We have uploaded the new supporting files to the system. We appreciate your professional guidance, and please contact us for any further revisions if there are other questions.

**Reviewer #1:**

This study examines 39,141 news articles published between 2010 and 2024 to understand the evolving media discourse surrounding HIV/AIDS. Employing Analysis of Topic Model Networks (ANTMN) and collocation analysis, we extract the thematic networks, terminology for people living with HIV (PLHIV), and HIV/AIDS metaphors. Multiple Correspondence Analysis (MCA) and network backbone method were complementarily employed to examine the interrelationships among discursive strategies and their connections with thematic contexts. In my opinion, this is an excellent paper characterized by rigorous logic, sound methodology, proficient English expression, reliable data, and credible conclusions. However, there are several minor issues that.

**Response:** We are grateful for the reviewer's valuable feedback. In the revised manuscript, we have highlighted the changes in yellow.

1. **Imprecise language expression:**

The phrase "particularly in contexts like China" is mentioned in lines 49–50. Please briefly elaborate on why China is specifically highlighted in this context.

**Response:** We appreciate reviewer’s inquiry about the specific mention of China. We have revised the text to clarify why China is specifically highlighted. We now note that HIV/AIDS has become the main cause of death in infectious diseases in China, and the number of high-risk groups is growing. In this case, it is particularly challenging to achieve the public health goal of eliminating HIV/AIDS. See lines 48-53 in Introduction.

2. **Formatting inconsistencies:**
   In line 74, two references are enclosed in square brackets with a comma between them, which does not comply with the formatting requirements; similar issues exist elsewhere in the text.
   An extra comma is present in line 133.
   The meaning of decimals and percentages next to the axes in Figures 3 and 4 is unclear—annotations should be provided to clarify these.

**Response:**

Thank you for pointing out these issues. We have carefully checked and revised the manuscript accordingly. The reference formatting (including the case in line 74 and similar instances elsewhere) has been corrected to comply with the journal’s style. The extra comma in line 133 has been removed. In addition, we have added annotations to Figures 3 and 4, respectively, to clarify the meaning of the axis labels. The numerical values represent the eigenvalues, and the percentages indicate the contribution rate of the corresponding dimension. The position of each point reveals its association with others within the two-dimensional semantic space. The complete annotations can be found in the captions of Figures 3 and 4. See lines 410-414 and 421-425 in Section 3.3

3. **Unclear research significance:**

It is recommended that the authors carefully elaborate, before the methodology section, on the significance of studying traditional media in the current era of highly prevalent social media. Specifically, please clarify: "What is the importance of researching traditional media when social media is so dominant?"

**Response:**

We appreciate reviewer’s insightful comment regarding the significance of studying traditional media in the age of social media. In the revised manuscript, we emphasize that traditional media still plays a central role in setting agendas and framing public perspective. We also highlight that traditional media discourse often provides reference points that other platforms react to or build upon. These additions, placed before the methodology section, clarify the research significance and justify our focus on traditional media. See lines 107-113 in Introduction.

4. **Superficial discussion on addressing deficiencies:**

While the paper identifies certain deficiencies in traditional media’s coverage of HIV-related topics, the discussion section only offers general remarks on how to improve these shortcomings. The authors are advised to provide targeted countermeasures and suggestions tailored to these specific deficiencies.

**Response:**

We appreciate the reviewer’s valuable comment. We agree that the original discussion of how to address deficiencies in traditional media coverage was too general and did not sufficiently translate our empirical findings into concrete, targeted recommendations. In the revised manuscript, we therefore added a paragraph that directly links the main deficiencies identified in our analysis to corresponding actionable recommendations. Specifically, we address the overemphasis on macro-level, policy-oriented narratives by recommending a rebalancing of thematic focus, creating regular spaces for the voices and lived experiences of PLHIV. We respond to the persistent and diversified use of stigmatizing labels by proposing that media organizations develop and disseminate a terminology guide with people-centered alternatives. We tackle the dominance and potential harms of war metaphors by encouraging a shift toward more constructive metaphors, combined with narratives emphasizing resilience, recovery. Finally, in light of the theme specific use of stigmatizing language, we emphasize the need for tailored interventions. These targeted suggestions have been incorporated into the Discussion section. See lines 586-600 in Section 4.2.

**Reviewer #2:** Firstly, this manuscript is technically sound and the data could support most part of the conclusion,and the statistical analysis has been performed relatively appropriately.

**Response:**

We are grateful for the reviewer's valuable feedback. In the revised manuscript, we have highlighted the changes in green.

1. However, for some part of data presented in the Result section, I couldn't find the further analysis and explanation in Discussion section. For example, when discussing the themes identified in this research, the fourth one "Medicine“ is not mentioned in "Key finding" part.

**Response:**

We are grateful for your positive feedback on our manuscript’s technical aspects and statistical analysis. We acknowledge that the "Medicine" theme, despite being identified as a primary theme, was not explicitly discussed in the "Key Findings" section. To address this, we have now incorporated a detailed discussion of the "Medicine" theme, including its thematic composition and temporal stability into the revised "4.1 Major Findings" section. See Section 4.1.

2. For another example, the second category of the result presented is "Changes over time", but I didn't find any further analysis and explanation on this in discussion section. I don't know whether it is one of the major findings of this research.

**Response:**

Thank you for your insightful comment. In the original manuscript, we did not sufficiently highlight the discussion of changes over time. In the revised version, we now discuss in more detail the trends and possible reasons for the changes in all five themes over time, including the rising prominence of “Prevention and control” and “Publicity,” the decline of “Society” and “PLHIV,” and the relative stability of “Medicine.” See Section 4.1.

3. Secondly, as far as I could see, all data collected in this research (including figures and tables) has been included at the end of the manuscript. Thirdly, as an academic paper, this manuscript is presented with the logical structure and in an intelligible fashion. The language is generally clear, correct and unambiguous, but as for the subtitle "Key Findings", "Major Findings" may be more accepted substitute.

**Response:** Thank you for your valuable suggestion. We have revised "Key Findings" to "Major Findings".
