# Supplementary material for: Constructing HIV/AIDS in Chinese media (2010–2024): A mixed-methods study
Source: PLoS One. 2026 May 13;21(5):e0349242. doi: 10.1371/journal.pone.0349242 (PMC13170867; doi:10.1371/journal.pone.0349242)
Supplement: Attachment — Submitted filename: reviewer attachments.docx [file pone.0349242.s003.docx]

Thank you for the opportunity to review this manuscript. The central limitation of this paper is its lack of a genuinely novel research contribution. Although the authors position the study as “enriching the interpretation of HIV/AIDS and social cognition in the Chinese context”, a substantial body of scholarship—both in Chinese and international communication studies—has already examined media framing, stigma construction, metaphor use, and agenda-setting in HIV reporting. From this perspective, the manuscript’s original contribution remains unclear. The findings largely reiterate established observations, such as the persistence of stigmatizing narratives and the dominance of macro-political frames. I would strongly encourage the authors to reconsider their research question and clarify what new insight this study offers beyond existing literature. At its current stage, the manuscript does not yet meet the standard for publication.

The framing of the research design is also problematic. The authors select 2011 as the starting point of analysis but do not provide a convincing justification for why this year constitutes a meaningful turning point in HIV communication or media practice. It is unclear whether this choice reflects a major policy shift, epidemiological change, or transformation in the media system. Moreover, the manuscript does not address the broader structural decline in the influence of traditional mainstream media in the digital era, which raises questions about the contemporary significance of focusing exclusively on such outlets. Without a clear rationale for the temporal scope and media selection, the analytical framework appears arbitrary rather than theoretically grounded.

The manuscript also demonstrates insufficient engagement with prior scholarship. Although earlier studies are briefly cited, the paper does not meaningfully situate its findings in relation to them. There is little discussion of whether the results confirm, challenge, or extend existing knowledge, nor is there a clear articulation of how this study advances debates in health communication, risk communication, or media discourse analysis. As a result, the research appears disconnected from the cumulative development of the field.

There are significant weaknesses in the methodological design and use of data. The study claims to analyse “media discourse construction,” yet the dataset consists primarily of news headlines containing HIV-related keywords. Headlines alone cannot adequately capture discursive structures, narrative strategies, source diversity, or contextual framing. This creates a mismatch between the stated research objectives and the empirical material. Furthermore, the manuscript characterises the approach as mixed-methods, but the analysis relies almost entirely on automated text mining techniques, with little evidence of qualitative validation, manual coding, or interpretive analysis. The methodological transparency and reliability of the findings are therefore difficult to assess.

Several of the paper’s central claims are insufficiently supported by evidence. Assertions regarding the persistence of stigma, the prevalence of war metaphors, and the dominance of political narratives are presented as major findings, yet these patterns have been widely documented in previous research. The manuscript does not demonstrate how the computational analysis produces substantively new insights or reveals patterns that could not have been identified through existing approaches. Consequently, the study remains largely descriptive and does not achieve the level of theoretical advancement expected for publication.

Finally, the manuscript does not clearly articulate its relevance for an international readership. While the empirical focus is on China, the discussion does not extract broader theoretical implications for global health communication or media studies. Without a clearer statement of how the findings contribute to international scholarship, the paper’s significance remains limited.

In summary, the manuscript addresses a potentially important topic but is constrained by insufficient originality, weak theoretical grounding, and limited engagement with prior research. Substantial reconceptualisation would be required for the study to make a meaningful scholarly contribution. For these reasons, I cannot recommend the manuscript for publication in its current form.
