# Supplementary material for: Constructing HIV/AIDS in Chinese media (2010–2024): A mixed-methods study
Source: PLoS One. 2026 May 13;21(5):e0349242. doi: 10.1371/journal.pone.0349242 (PMC13170867; doi:10.1371/journal.pone.0349242)
Supplement: Attachment — Submitted filename: Response_to_Reviewers_auresp_2.docx [file pone.0349242.s004.docx]

**Dear Editor,**

Thank you for your prompt review of our manuscript. We deeply appreciate the constructive and insightful comments provided by both you and the reviewers. This feedback has significantly strengthened the clarity, rigor, and theoretical depth of our research, and we have carefully revised the manuscript to address all points raised.

1. First, you need to clearly articulate the novel contribution of the study. While your large-scale (39,141 articles) and long-term (15-year) analysis has the potential to offer new insights, the current manuscript fails to explicitly contrast your findings with prior research. Please highlight the unique discoveries of your study, such as the identification of understudied stigmatizing terminologies (e.g., spatialized terms like “AIDS Town” and “AIDS Apartment”) and the novel analysis of the associations between thematic contexts, PLHIV (people living with HIV) terminologies, and HIV/AIDS metaphors. Additionally, explain how these findings fill gaps in the existing literature.

**Response:**

Thank you for this constructive feedback. We have significantly revised Section 4.1 to clearly elaborate on our novel contributions and have added an additional paragraph comparing our findings with previous research.

Specifically, we contrasted our findings with earlier studies to highlight the evolution of spatial stigma, demonstrating how stigma has adapted from rural contexts (e.g., "AIDS Village") to urbanized and modern social structures (e.g., "AIDS Apartment" ).

Furthermore, we clarified the novelty of our multidimensional analysis. We emphasized that, unlike previous studies that analyzed themes and language in isolation, our intersectional approach reveals how macro-level contexts dictate micro-level linguistic choices, thereby uncovering deeper biases like the gendered narratives within the "Society" theme.

Finally, a new concluding paragraph highlights the methodological advantage of our 15-year computational dataset. We explained how it successfully detects latent, low-frequency discursive shifts—such as the active emergence of positive "journey" and "entity" metaphors—that traditional manual sampling often overlooks. We believe these revisions significantly strengthen the articulation of the paper's core contributions. **Please see the highlighted part in 4.1.**

2. Next, please provide a convincing explanation for selecting 2011 as the starting point of your analysis. Clarify whether this year corresponds to a major policy shift, epidemiological change, or transformation in the Chinese media system. Address the broader structural decline in the influence of traditional mainstream media in the digital era, and clearly explain the contemporary significance of focusing exclusively on traditional media outlets in your study.

**Response:**

Thank you for your valuable feedback. First, we would like to apologize for a typo in the title of our previous manuscrip: our analysis actually begins in 2010, not 2011. We have corrected this throughout the text. We have expanded Section 2.1 (Data Collection) to justify this starting point based on three critical shifts in 2010: 1) Epidemiological: Sexual transmission overtook other routes as the primary infection mode; 2) Policy: China lifted its entry ban on foreigners living with HIV; and 3) Media: The rapid rise of social media began transforming China's media ecology. **Please see Section 2.1, Paragraph 2, Lines 149-162.**

Thank you for raising this important point regarding the structural decline of traditional media in the digital era. To address this, we have added content to the fourth paragraph of Section 1 explaining that while social media has indeed fragmented audiences, focusing on traditional mainstream media remains methodologically and contextually critical. **Please see lines 107-115 in Section 1.**

3. The current manuscript only briefly cites earlier studies but does not meaningfully situate your findings in relation to them. Please add a section in the discussion to explicitly discuss whether your results confirm, challenge, or extend existing knowledge, and clearly articulate how this study advances debates in health communication, risk communication, or media discourse analysis.

**Response:**

We sincerely thank the reviewer for this constructive suggestion. In response, we have expanded Section 4.1 to explicitly situate our work within the broader literature, detailing how our findings both challenge and extend existing knowledge. Specifically, the new text explains how our computational approach extends current theoretical frameworks by uncovering latent stigmatizing patterns that permeate modern social structures, moving beyond traditional research focused on marginalized groups. Additionally, we explicitly note how our results challenge prevailing narratives in Chinese HIV/AIDS discourse by highlighting active destigmatization efforts and the emergence of positive metaphors that prior studies have largely obscured. Together, these additions clearly articulate how our longitudinal analysis advances ongoing debates in health communication and media discourse. **Please see the last paragraph of Section 4.1, lines 610-633.**

Furthermore, to explicitly articulate our study's contributions to the specific domains you highlighted, we have substantially revised the first paragraph of Section 4.2. The updated text now clearly delineates how our findings: (1) illuminate the dynamics of a state-dominated, policy-driven governance system within health communication; (2) capture the paradigm shift from adversarial to constructive frames in risk communication; and (3) utilize our large-scale computational approach to push media discourse analysis beyond traditional descriptive framing by detecting latent, low-frequency stigmatizing terminologies. **Please see lines 636-652 in the first paragraph of Section 4.2.**

4. Additionally, please clarify that your study analyzes the full text of news articles (not just headlines) that contain HIV-related keywords, to eliminate any misunderstanding from the reviewer.

**Response:**

We thank the reviewer for pointing out this potential ambiguity. To ensure complete clarity, we have revised Section 2.1 to explicitly state that while our initial search criteria collected news articles based on HIV-related keywords in their headlines, all subsequent computational analyses were conducted on the full texts of these articles. We believe this addition eliminates any misunderstanding regarding the scope of our corpus. **Please see lines 145-147 in 2.1.**

5. Complement your automated text mining techniques with more qualitative validation, such as manual coding and interpretive analysis of a subset of articles, to enhance the reliability and depth of your findings.

**Response:**

We sincerely thank the reviewer for this valuable suggestion. To enhance the reliability and depth of our findings, we have complemented our automated text mining with targeted qualitative validation. As detailed in the revised Section 2.4, two independent researchers conducted manual coding and interpretive analysis on a subset of articles, specifically focusing on the intersection of "Children" terminology and entity metaphors. We have incorporated the results of this manual annotation into Section 3.4, supported by Table 4 that outlines the source elements of these metaphors. Finally, we expanded Section 4.1 to discuss these qualitative insights, demonstrating how the dominant use of terms like *xiedai* (carry) unintentionally amplifies societal stigma by framing vulnerable children as active, mobile vectors rather than patients in need of care. **Please see Section 2.4, Lines 278–290; Section 3.4, Lines 473–487; and Section 4.1, Lines 598-609.**

6. Although your empirical focus is on China, the current discussion does not extract broader theoretical implications for global health communication or media studies. Please add a section to explain how your findings contribute to international scholarship, and discuss the potential transferability of your results to other contexts.

**Response:**

We appreciate this valuable suggestion. In the revised Section 4.2, we added a discussion on the broader theoretical implications and global transferability of our findings. Specifically, we demonstrate how the shift from confrontational to constructive metaphors offers a predictive framework for analyzing media discourse on other infectious diseases transitioning to normalized management. Additionally, we explain how the expansion of stigma terminology into modern social and urban structures provides a transferable analytical framework for understanding how evolving socioeconomic conditions reshape stigma language across different cultural contexts. **Please see the last paragraph of Section 4.2, lines 677-691.**

7. Finally, demonstrate how your computational analysis produces substantively new insights or reveals patterns that could not have been identified through existing approaches, and move beyond descriptive analysis to achieve a higher level of theoretical advancement.

**Response:**

We appreciate this valuable feedback. In the revised Section 4.1, we explicitly highlight how our computational approach achieves theoretical advancement by moving beyond traditional descriptive statistics. Specifically, this methodology enabled the detection of highly latent, low-frequency discursive patterns that conventional manual sampling would inevitably overlook, allowing us to produce substantively new insights into the structural permeation of stigma in modern society. **Please see lines 614-618 in 4.1.**

**Reviewer #3:**

**1.** Please check the formatting throughout. Some of the paragraphs too long and really hard for the reader to read.

**Response:**

We thank the reviewer for this helpful feedback regarding readability. We have carefully reviewed the formatting throughout the entire manuscript and broken down lengthy paragraphs into shorter, more focused segments.

**2.** Suggest to put why 14 years of study because the duration of the publications used too old and dynamic of the situations not really addressed here. Maybe 10 years is still ok.

**Response:**

We sincerely thank the reviewer for prompting us to clarify the rationale behind our study's timeframe. In the revised manuscript, we have explicitly justified why a 15-year span starting in 2010 is essential for capturing the complete dynamic evolution of this discourse, which a shorter 10-year window would omit. Specifically, we detail three critical turning points that occurred in 2010: the epidemiological shift to sexual transmission as the primary infection mode, the policy milestone of lifting the entry ban on foreigners living with HIV, and the fundamental transformation of China's media ecology driven by the rise of social media. We believe these additions clearly articulate the significance of the 2010 starting point and effectively address the concern regarding the situational dynamics of our selected period. **Please see Section 2.1, Paragraph 2, Lines 149-162.**

**3.** The transformation of issues in current media (social media or current policies) should also addressed here.

**Response:**

We thank the reviewer for highlighting the need to address the evolving media landscape. In response, we have added text clarifying why focusing on mainstream traditional media remains methodologically crucial for our 15-year longitudinal study, despite the rise of social media. Specifically, we explain that traditional outlets retain unparalleled institutional authority as barometers for state policy and serve as foundational agenda-setters whose authoritative discourses are subsequently amplified online. Furthermore, unlike the ephemeral and algorithmically biased nature of social media data, traditional media provides a stable, archived textual record, ensuring the methodological robustness of our long-term analysis. **Please see lines 107-115 in Section 1.**

**Reviewer #4:**

**1.** The Discussion section fails to fully address the research gaps outlined in the Introduction, nor does it establish a systematic, multi-dimensional critical dialogue and comparative analysis with the classic domestic and international studies and cutting-edge field-specific research reviewed in the same section.

**Response:**

We sincerely thank the reviewer for this insightful critique. To establish a systematic and multi-dimensional dialogue with existing scholarship, we have substantially expanded the Discussion section. The newly added text directly addresses the research gaps outlined in the Introduction by explicitly comparing our findings with prior studies. Specifically, we contrast our computational discovery of latent, structural stigmas with traditional literature that primarily focuses on marginalized groups. Furthermore, we challenge prevailing academic narratives by highlighting the emergence of positive metaphors and exposing multidimensional narrative biases, moving well beyond the scope of classic univariate studies and traditional negative framing. We hope this expanded comparative analysis helps to better situate our findings within the broader academic discourse. **Please see the highlighted part in 4.1.**

**2.** The manuscript does not proactively acknowledge its core limitations, including sampling frame bias, the omission of media heterogeneity analysis, and subjectivity in coding procedures, which compromises the overall academic rigor of the study.

**Response:**

We sincerely thank the reviewer for highlighting the need for a more critical reflection on our methodology. To improve the study's academic rigor, we have revised Section 4.3. This revised section explicitly acknowledges our core constraints: the potential for sampling frame bias given our focus on specific traditional media outlets, the lack of media heterogeneity analysis regarding diverse social media narratives , and the inherent subjectivity and limitations of our collocation-based coding procedures. We hope this transparent discussion effectively strengthens the overall rigor of the manuscript. **Please see Section 4.3.**

**3.** The proposed recommendations for future research are overly broad, and do not put forward specific, extensible research directions closely aligned with the research gaps identified in this paper.

**Response:**

We thank the reviewer for this constructive feedback. In the revised Section 4.3, we replaced our previous broad statements with specific, actionable future research directions aligned with our study's identified gaps.

Specifically, we now propose three targeted avenues: (1) expanding the sampling frame to compare narratives across diverse traditional media types; (2) conducting cross-media analyses to track stigmatizing frames between traditional outlets and social media platforms; and (3) utilizing deep learning and large language models to automatically detect complex, implicit stigmatization beyond predefined lexicons. We hope these concrete recommendations provide clearer pathways for future scholarship. **Please see Section 4.3.**

**Reviewer #5:**

1. The central limitation of this paper is its lack of a genuinely novel research contribution. Although the authors position the study as “enriching the interpretation of HIV/AIDS and social cognition in the Chinese context”, a substantial body of scholarship—both in Chinese and international communication studies—has already examined media framing, stigma construction, metaphor use, and agenda-setting in HIV reporting. From this perspective, the manuscript’s original contribution remains unclear. The findings largely reiterate established observations, such as the persistence of stigmatizing narratives and the dominance of macro-political frames. I would strongly encourage the authors to reconsider their research question and clarify what new insight this study offers beyond existing literature. At its current stage, the manuscript does not yet meet the standard for publication.

**Response:** We sincerely thank the reviewer for this highly constructive critique. We have substantially revised Section 4.1 to explicitly contrast our findings with prior research. Unlike previous studies that largely focus on traditional marginalized groups and negative framing, our 15-year computational approach uncovers highly latent, low-frequency discursive shifts. These include the evolution of stigma into modern urban spaces (e.g., "AIDS Apartment") and the active emergence of positive "journey" and "entity" metaphors. We hope these additions, along with our multidimensional analysis of narrative biases, provide a clearer context for the theoretical insights our study aims to offer **(Please see highlighted parts in Section 4.1)**.

2. The framing of the research design is also problematic. The authors select 2011 as the starting point of analysis but do not provide a convincing justification for why this year constitutes a meaningful turning point in HIV communication or media practice. It is unclear whether this choice reflects a major policy shift, epidemiological change, or transformation in the media system. Moreover, the manuscript does not address the broader structural decline in the influence of traditional mainstream media in the digital era, which raises questions about the contemporary significance of focusing exclusively on such outlets. Without a clear rationale for the temporal scope and media selection, the analytical framework appears arbitrary rather than theoretically grounded.

**Response:**

We deeply appreciate your meticulous reading and entirely agree with your concern regarding the justification of our temporal and spatial scope. First, we sincerely apologize for a typo in the title

of our previous manuscript.: our study actually begins in 2010, not 2011. We have corrected this and expanded Section 2.1 to justify this starting point based on three 2010 milestones: the epidemiological shift to sexual transmission, the lifting of China's entry ban on foreigners with HIV, and the dawn of the social media era. Furthermore, we expanded Section 1 to explain that despite social media's rise, traditional mainstream media remains methodologically crucial for a longitudinal study because it provides stable, institutionally archived records and serves as the foundational agenda-setter **(Please see lines 107-115 and 149-162)**.

3. The manuscript also demonstrates insufficient engagement with prior scholarship. Although earlier studies are briefly cited, the paper does not meaningfully situate its findings in relation to them. There is little discussion of whether the results confirm, challenge, or extend existing knowledge, nor is there a clear articulation of how this study advances debates in health communication, risk communication, or media discourse analysis. As a result, the research appears disconnected from the cumulative development of the field.

**Response:**

We sincerely thank the reviewer for this constructive suggestion. In response, we have expanded Section 4.1 to explicitly situate our work within the broader literature, detailing how our findings both challenge and extend existing knowledge. Specifically, the new text explains how our computational approach extends current theoretical frameworks by uncovering latent stigmatizing patterns that permeate modern social structures, moving beyond traditional research focused on marginalized groups. Additionally, we explicitly note how our results challenge prevailing narratives in Chinese HIV/AIDS discourse by highlighting active destigmatization efforts and the emergence of positive metaphors that prior studies have largely obscured. Together, these additions clearly articulate how our longitudinal analysis advances ongoing debates in health communication and media discourse **(Please see the last paragraph of Section 4.1, lines 610-633).**

Furthermore, to explicitly articulate our study's contributions to the specific domains you highlighted, we have substantially revised the first paragraph of Section 4.2. The updated text now clearly delineates how our findings: (1) illuminate the dynamics of a state-dominated, policy-driven governance system within health communication; (2) capture the paradigm shift from adversarial to constructive frames in risk communication; and (3) utilize our large-scale computational approach to push media discourse analysis beyond traditional descriptive framing by detecting latent, low-frequency stigmatizing terminologies **(Please see lines 636-652 in the first paragraph of Section 4.2).**

4. There are significant weaknesses in the methodological design and use of data. The study claims to analyse “media discourse construction,” yet the dataset consists primarily of news headlines containing HIV-related keywords. Headlines alone cannot adequately capture discursive structures, narrative strategies, source diversity, or contextual framing. This creates a mismatch between the stated research objectives and the empirical material. Furthermore, the manuscript characterises the approach as mixed-methods, but the analysis relies almost entirely on automated text mining techniques, with little evidence of qualitative validation, manual coding, or interpretive analysis. The methodological transparency and reliability of the findings are therefore difficult to assess.

**Response:**

We are deeply grateful to the reviewer for pointing out these critical methodological ambiguities. First, we sincerely apologize if our original phrasing was severely lacking in clarity and caused this misunderstanding. To clarify, our analysis was actually conducted on the full texts of the news articles, not just the headlines. We have now explicitly stated in Section 2.1 that while headlines were used for initial screening, all computational and interpretive analyses utilized full texts.

Second, as you rightly pointed out, our study leans heavily toward computational analysis. We initially used the term "mixed-methods" to reflect the integration of multiple independent analytical techniques (such as topic modeling and metaphor identification) within a framework that combines human expert knowledge with computational efficiency. Nevertheless, we completely agree with your insightful suggestion that adding qualitative validation would address this methodological limitation and greatly enhance the study. Therefore, in the latest revision, we have introduced targeted qualitative validation to complement our text mining. As detailed in Sections 2.4 and 3.4, two independent researchers conducted manual coding and interpretive analysis on a subset of articles, with results summarized in the new Table 4 **(Please see Section 2.4, Lines 278–290; Section 3.4, Lines 473–487; and Section 4.1, Lines 598-609)**.

5. Several of the paper’s central claims are insufficiently supported by evidence. Assertions regarding the persistence of stigma, the prevalence of war metaphors, and the dominance of political narratives are presented as major findings, yet these patterns have been widely documented in previous research. The manuscript does not demonstrate how the computational analysis produces substantively new insights or reveals patterns that could not have been identified through existing approaches. Consequently, the study remains largely descriptive and does not achieve the level of theoretical advancement expected for publication.

**Response:**

This is an exceptionally fair and penetrating critique. We fully acknowledge that findings such as the persistence of stigma and war metaphors are indeed well-documented, and merely restating them offers little theoretical value. We have updated Section 4.1 to explicitly demonstrate how our computational methodology achieves theoretical advancement beyond descriptive statistics. Specifically, this approach enabled the detection of highly latent, low-frequency patterns—such as previously undocumented spatial and social role stigmas (e.g., "AIDS Teacher")—that conventional manual sampling would inevitably overlook. This large-scale detection provides substantively new insights into the structural permeation of stigma that existing approaches could not easily identify **(Please see lines 614-618 in 4.1)**.

6. Finally, the manuscript does not clearly articulate its relevance for an international readership. While the empirical focus is on China, the discussion does not extract broader theoretical implications for global health communication or media studies. Without a clearer statement of how the findings contribute to international scholarship, the paper’s significance remains limited.

**Response:**

We thank the reviewer for highlighting this gap. Thanks to your prompt, we have critically reflected on the global transferability of our findings. We have added a discussion in Section 4.2 to extract the broader global implications of our work. We demonstrate how our findings on the shift from confrontational to constructive metaphors offer a predictive framework for media discourse on other infectious diseases transitioning to normalized management globally. We also explain how our spatial stigma framework can be transferred to understand evolving socioeconomic conditions in other cultural contexts **(Please see the last paragraph of Section 4.2, lines 677-691)**.

7. In summary, the manuscript addresses a potentially important topic but is constrained by insufficient originality, weak theoretical grounding, and limited engagement with prior research. Substantial reconceptualisation would be required for the study to make a meaningful scholarly contribution. For these reasons, I cannot recommend the manuscript for publication in its current form.

**Response:**

Thank you once again for your incredibly helpful and detailed review. Your guidance was instrumental in helping us refine our theoretical focus and methodological transparency. We are truly grateful for the opportunity to improve our work based on your insights, and we hope this revised manuscript now meets the high standards of the journal.
