# Supplementary material for: Constructing HIV/AIDS in Chinese media (2010–2024): A mixed-methods study
Source: PLoS One. 2026 May 13;21(5):e0349242. doi: 10.1371/journal.pone.0349242 (PMC13170867; doi:10.1371/journal.pone.0349242)
Supplement: Attachment — Submitted filename: Response_to_Reviewers_auresp_3.docx [file pone.0349242.s005.docx]

Thank you for your revised manuscript and detailed responses to the reviewers. The manuscript has been substantially improved and is now close to acceptance. However, several specific, fixable errors must be corrected before final acceptance. Below are the required revisions with clear references to the manuscript text.

We would like to express our sincere gratitude to the editor for the constructive feedback and support throughout the review process. We have used yellow highlighting to indicate corrections for inconsistencies, grammar, and formatting errors, and blue highlighting for revisions in the methodology section and table notes.

1.Grammatical and subject-verb agreement errors
Abstract: “stigmatizing terminology persist”
Introduction: “one of the leading cause”
Persistent issues with verb number, noun number, and awkward phrasing appear throughout the manuscript.

**Response**:We sincerely thank the editor for this careful reading. We have conducted a thorough, systematic revision of the entire manuscript to address all grammatical and subject-verb agreement errors.

Please refer to the highlighted yellow parts.

2.Inconsistent terminology and labeling
Inconsistent reference to affected populations: PLHIV, people living with HIV, HIV patients, AIDS patients are used interchangeably.
Inconsistent pluralization: metaphor / metaphors, journey metaphor / journey metaphors vary across sections.

**Response**: We thank the editor for this observation. We have systematically standardized all terminology throughout the revised manuscript. For references to affected populations, we have adopted "PLHIV" (people living with HIV) as the sole standard term, with the full form at first mention and the abbreviation thereafter. For the disease name, we have unified all instances to "HIV/AIDS" throughout the manuscript, replacing inconsistent variants such as "AIDS," "HIV," or "the virus" when referring to the disease as a whole. It should be specifically noted that the original Table 1 contained phrases such as "AIDS Patient" and "HIV-infected Person," which were actually direct translations of Chinese terms from Chinese news sources, rather than inconsistencies in terminology. If these phrases in Table 1 were also changed to "PLHIV," the differences reflected in the Chinese examples would not be captured. Therefore, in the revised version of Table 1, we have retained these phrases while also adding the corresponding pinyin of the Chinese terms to indicate that these phrases are translations of Chinese terms, rather than the chosen terminology of this paper. For metaphors, we reviewed and standardized the singular and plural forms used in different instances throughout the paper. Please refer to the highlighted yellow parts.

Please refer to the highlighted yellow part, which involves metaphors, PLHIV, and HIV/AIDS.

3.Conflicting numerical values across sections
Sample size: 40,038 raw; 39,141 valid in Methods, but inconsistent rounding appears in the Abstract, cover letter, and responses.

**Response**: To address the concern regarding inconsistent rounding of the sample size, we have removed the specific count (39,141) from the Abstract, replacing it with a qualitative description. This eliminates potential rounding conflicts across different sections of the manuscript. The exact sample size (40,038 raw; 39,141 valid) is now presented exclusively in the Methodology section as the singular reference point for all statistical calculations. All approximate or inconsistently rounded figures throughout the main text, cover letter, and responses have been cross-checked and harmonized to ensure internal consistency and transparency.

Please refer to the blue highlighted part of the Abstract.

Topic number: 50 topics during modeling vs. 48 topics in Results; these values must be aligned.

**Response**: We thank the reviewer for pointing out the need for clarity regarding the topic numbers. In accordance with the standard procedures for Analysis of Topic Model Networks (ANTMN) , we initially identified 50 topics using the LDA model to ensure comprehensive coverage of the corpus. Following the methodology established by Walter and Ophir (2019), we qualitatively evaluated all 50 topics and excluded 2 "boilerplate" topics that lacked substantive semantic meaning or were composed of purely administrative/template language. This resulted in a refined set of 48 topics used for all subsequent analytical steps. To prevent any further ambiguity, we have revised the "Methodology" section to explicitly state that while 50 topics were initially generated, the analysis consistently proceeds with the 48 validated topics. We have also cross-verified all figures, tables, and text in the "Results" and "Discussion" sections to ensure they uniformly report the final count of 48 topics.

Ophir Y. News frame analysis: an inductive mixed-method computational approach. Commun Methods Meas. 2019;13(4):248-66. doi: 10.1080/19312458.2019.1639145.

Please refer to the blue highlighted part of the Topic network.

4.Insufficient methodological transparency
Mixed-methods validation: No explicit number of articles used for qualitative coding, no coder details, and no exact Krippendorff’s Alpha value reported.

**Response**: We sincerely appreciate the reviewer’s feedback regarding the transparency of our qualitative analysis. We have now provided comprehensive details of the coding process in the revised "Methodology" section to address these omissions.

Please refer to the blue highlighted part of the Collocation extraction and Co-occurrence pattern.

Multiple Correspondence Analysis (MCA): No eigenvalues or dimension contribution rates reported in the results.

**Response**: We appreciate the professional suggestion regarding the reporting of Multiple Correspondence Analysis (MCA) results. We have now included the missing statistical parameters in the "Results" section to enhance the transparency and rigor of our analysis.

Please refer to the blue highlighted part of the PLHIV terminology and HIV/AIDS metaphors.

5.Incomplete result presentation and unclear tables
Table 4 lacks clear annotation, sample size, and coding rules.

**Response**: We sincerely appreciate this suggestion. We have added a clear annotation to Table 4. Additionally, in the new revised version, we have included more detailed descriptions regarding the sample size and coding rules in both the Methodology and Results sections.

Please refer to the blue highlighted part of the Table 4.

Usage rates in Tables 1 and 2 show inconsistent decimal places.

**Response**: We appreciate the reviewer's comment regarding the decimal places. In the original version, we used '< 0.01%' to avoid the misleading '0.00%' for extremely low frequencies. However, this led to the simultaneous presence of '0.01%' (rounded from values like 0.014%) and '< 0.01%' (truncated from values below 0.005%), creating a visual inconsistency in precision.

In the revised manuscript, we have standardized the reporting strategy for both Table 1 and Table 2. We now use '< 0.05%' as a unified threshold for all values below this level. All other usage rates are strictly formatted to two decimal places (e.g., 1.00%). This adjustment ensures a consistent visual standard and statistical rigor across all tables.

Finally, we have thoroughly reviewed and revised all numerical percentages within the main text to ensure that the number of decimal places is consistent throughout the entire manuscript.

Please refer to the blue highlighted part of the Table 1 and 2.

MCA results lack sufficient statistical supporting information.

**Response**: We appreciate your professional suggestion. In the revised Results section, we have provided a more detailed description and interpretation of the MCA.

Please refer to the blue highlighted part of the PLHIV terminology and HIV/AIDS metaphors.

6.Formatting and presentation flaws
References show inconsistent DOI formatting, journal abbreviations, and citation style.
Subheading numbering is disorganized; some paragraphs remain overly long.
Unremoved revision traces and inconsistent line spacing are present.

**Response**: We sincerely thank the editor for pointing out these formatting issues. In the revised manuscript, we have thoroughly standardized the references (including DOI formats and journal abbreviations) to ensure a consistent citation style. Additionally, we revised the formatting of the headings in accordance with the journal's style guide to make them clearer. Finally, we have divided overly long paragraphs to improve readability, removed all previous revision traces, and unified the line spacing throughout the document to strictly adhere to the journal's formatting guidelines.

Please refer to the highlighted yellow parts.
